# Circulating Tumor DNA from Ascites as an alternative to tumor sampling for genomic profiling in ovarian cancer patients

**DOI:** 10.1186/s40364-023-00533-1

**Published:** 2023-10-20

**Authors:** Maria Kfoury, Reda El Hazzaz, Claire Sanson, Felix Blanc Durand, Judith Michels, Emeline Colomba Blameble, Roseline Tang, Audrey Le Formal, Elodie Lecerf, Sebastien Gouy, Amandine Maulard, Patricia Pautier, Etienne Rouleau, Alexandra Leary

**Affiliations:** 1 Department of Oncology, Gustave Roussy Cancer Center, Villejuif, 94800 France; 2 Department of Oncology, Institut Paoli-Calmettes, 232 Boulevard Sainte Marguerite, Marseille, 13009 France; 3 Department of Medical Oncology, AR-RAZI Cancer Center, FEZ, Villejuif, 30050 Morocco; 4 Department of Surgery, Hôpital Pitié-Salpétrière, Paris, 75013 France; 5 Department of Medical Biology and Pathology, Cancer Genetics Laboratory, Villejuif, 94800 France; 6 Inserm UMR 981, Gustave Roussy Cancer Center, Villejuif, 94800 France; 7 Department of Surgery, Gustave Roussy Cancer Center, Villejuif, 94800 France

**Keywords:** Circulating Tumor DNA, Ovarian cancer, Homologous recombination deficiency, Genomic instability score, Ascites

## Abstract

Genomic testing is crucial for the management of ovarian cancer. DNA from biopsies at diagnostic laparoscopies or interval debulking surgery after neoadjuvant chemotherapy, has a high failure rate. At relapse, biopsies may not be feasible. The aim of our study was to evaluate the feasibility and usefulness of measuring genomic instability score (GIS) on cell-free DNA (cfDNA) from ascites.

Patients enrolled in a prospective study (NCT03010124) consented to analysis of biological samples. CfDNA was extracted from 1 to 4 ml of double-centrifuged fresh ascites. Targeted Next-generation sequencing (NGS) including *TP53* mutation (*TP53m*) was performed on cfDNA to confirm the presence of tumor cfDNA. Single Nucleotide Polymorphism Array estimating somatic copy number alterations (SCNA) was performed to calculate GIS for Homologous-Recombination deficiency (HRD).

Twenty nine ascites were collected from 20 patients with suspected or confirmed OC. 93% (27/29) samples had detectable cfDNA (median 1120 ng [24-5732]) even when obtained during chemotherapy. A deleterious mutation was identified in 100%, with high allelic frequencies (median 60% [3.3–87%]), confirming that cfDNA was tumoral. SCNA analyses on 17 patients showed 11 high GIS, and 6 low GIS. 4 patients with confirmed BRCA mutation had a high GIS on ascites. When available from the same patient, SCNA profiles on ascites and tumor were superimposable.

Ascites is frequent at diagnosis and relapse and yields large amounts of tumoral cfDNA. SCNA analysis on ascitic cfDNA is feasible and can detect the same HRD scar as tumor testing. Ascites could provide an alternative to tumor sampling for HRD and BRCA testing.

**Supplementary Information:**

The online version contains supplementary material available at 10.1186/s40364-023-00533-1.

## Main text

To the editor,

Genomic testing is crucial for the management of ovarian cancer (OC). Approximately 25% of high-grade OC have germline or somatic *BRCA1* or *BRCA2* mutations [1]. 50% of high-grade OC are homologous recombination deficient (HRD). HRD is defined by the detection of a *BRCA1 or BRCA2 mutation*, or demonstration of high genomic instability, that can be measured by Genomic Instability Scores (GIS) [2]. These defects in homologous recombination increase tumor sensitivity to platinum chemotherapy and PARP inhibitors [3, 4]. 20% of patients with advanced OC, have non-contributive HRD testing on FFPE tumor based assays [5], often attributable to small biopsies obtained during upfront or interval debulking surgery. New radiologically guided biopsies are often not technically or safely feasible. In the last decade, the utility of liquid biopsies for cancer management has been demonstrated in several studies [6–8]. High-grade OC are characterized by almost universal somatic *TP53* mutations (*TP53m*) which may be particularly suited to this approach [9].

In our study, we aimed to investigate the feasibility and clinical utility of tumor derived cfDNA from ascitic samples, as an alternative to tissue biopsy, to measure genomic instability as a surrogate for HRD.

Patients enrolled in a prospective study (NCT03010124) consented to analysis of biological samples. CfDNA was extracted from fresh ascites. Targeted Next-generation sequencing (NGS) including *TP53m* and SNParray for somatic copy number alterations (SCNA) analyses to calculate GIS were performed. Detailed material and methods are described in the supplementary material.

**Fig. 1:**
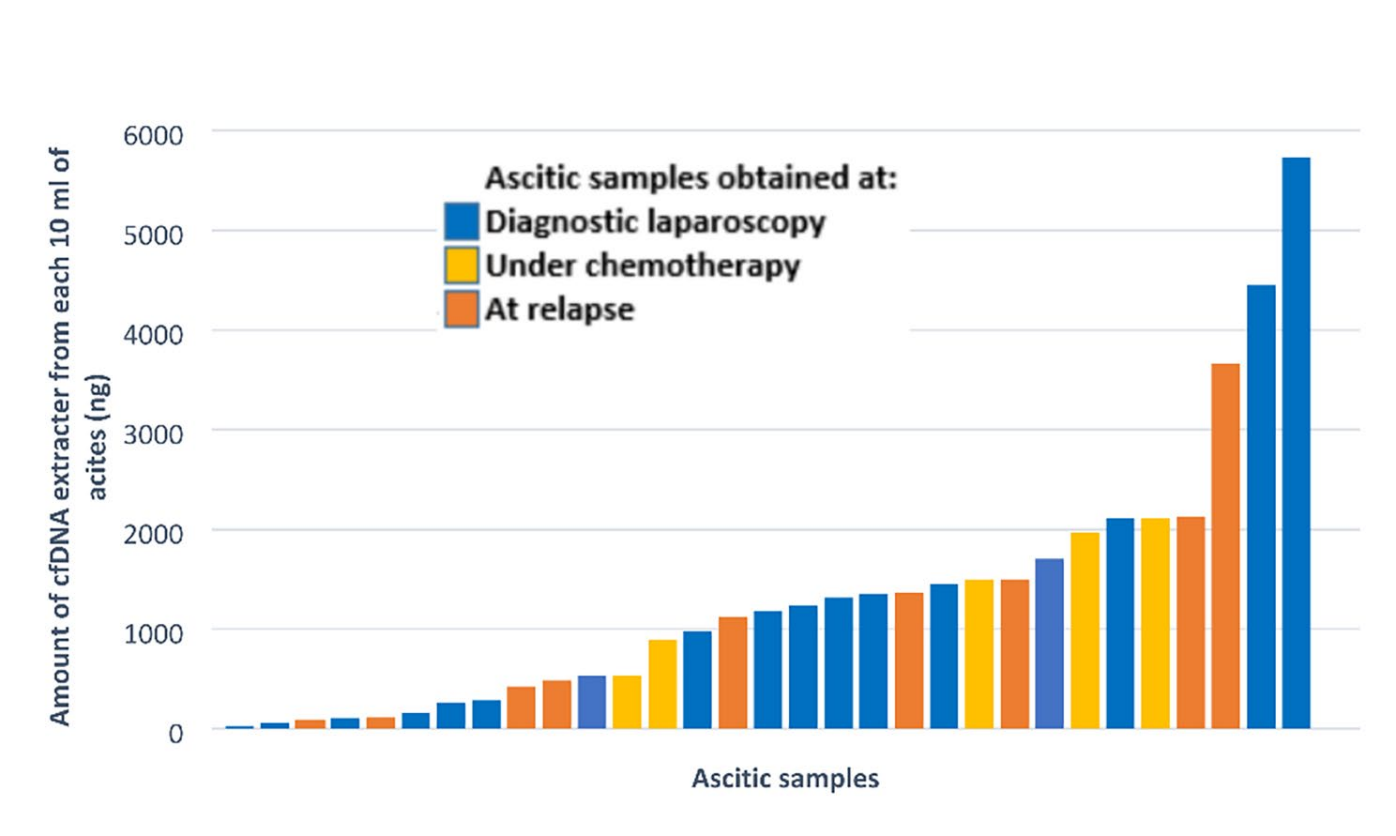
High yield of tumoral cell-free DNA in ascites Abbreviations: cfDNA: cell-free DNA

Overall, 29 ascites were collected from 20 patients with high-grade OC from March 2015 to January 2016. Ten ml of ascites were double centrifuged for cfDNA extraction. All samples (100%) had detectable cfDNA (median 1120 ng, range: 24-5732 ng) (Fig. 1).

**Fig. 2:**
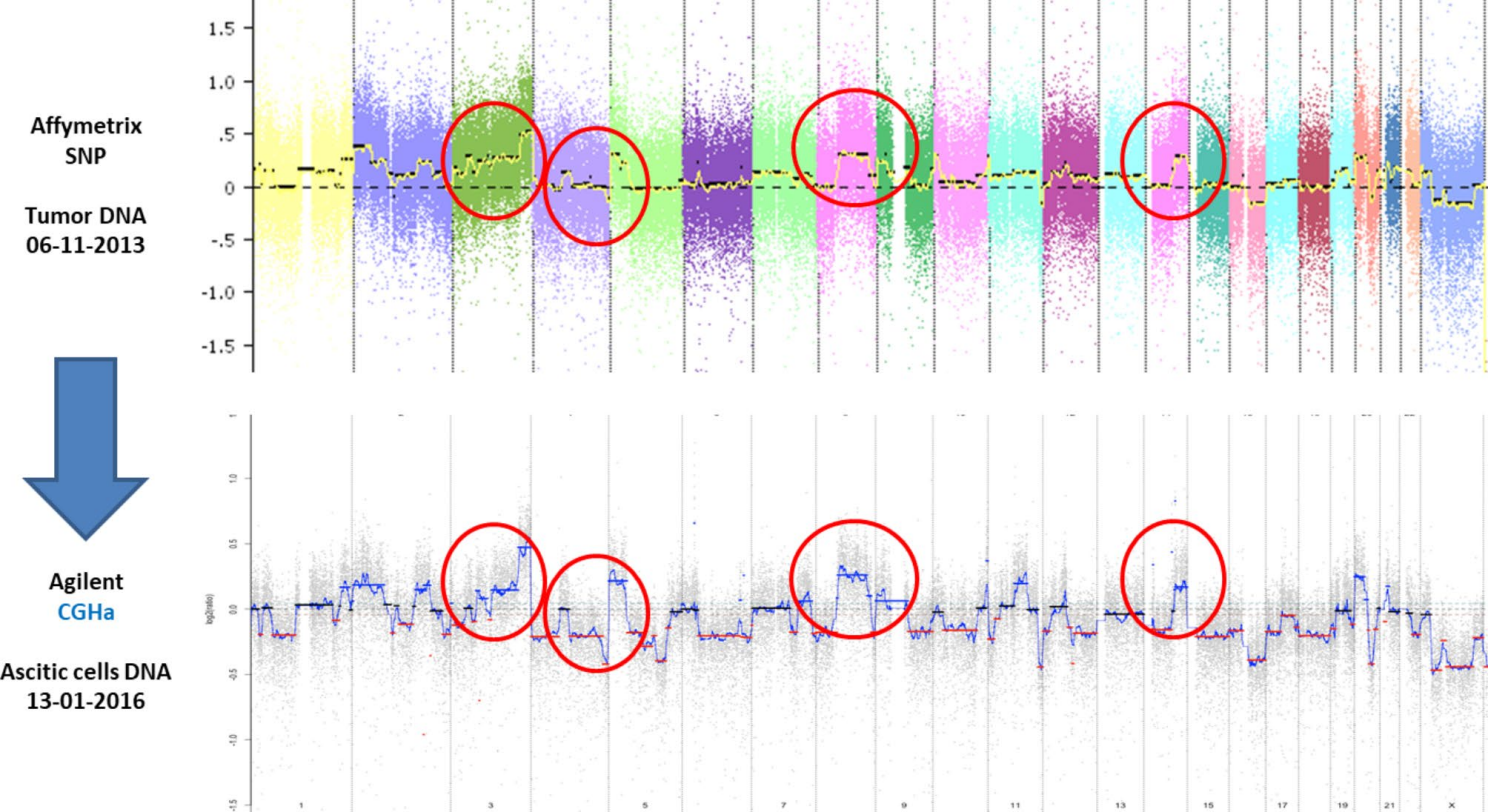
Comparison between SCNA profiles derived from tumor tissue and circulating tumor DNA Abbreviations: CGHa: Comparative Genomic Hybridization Array, SCNA: Somatic Copy Number Alteration, SNP: Single Nucleotide Polymorphism Array

In 27/29 (93%) samples, a deleterious mutation was identified with high median allelic frequency: 60% (range 4–87%). Among 27 samples with confirmed cfDNA, a *TP53m* was detected in 25 (92%) samples confirming cfDNA is tumoral (Supplementary Table 2). We performed SCNA on cfDNA from ascites from patients with high-grade OC. Analysis were contributive and GIS was feasible on 17/20 (85%) patients. Samples were classified as high versus low genomic instability. 11/17 patients had a high GIS and were considered as homologous recombination deficient (HRD), and 6 patients a low GIS. All 5 patients with known germinal BRCA1/2 mutation included in this study had a high GIS score on ascites. When both were available from the same patient, the SCNA profiles derived from ctDNA in ascites and tumor sampling were superimposable (Fig. 2).

To determine the proportion of patients with sufficient ascites for ctDNA analysis, we performed a retrospective review of the operative reports from 100 consecutive patients who underwent a diagnostic and interval laparoscopy for high-grade OC. 97/100 patients had visible peritoneal fluid: volume of ascites ranged from 50 to 11,000 ml, with a median of 500 ml. After neoadjuvant chemotherapy, 40% of patients still had ascites with a volume ranging from 80 to 5000 ml, median of 150 ml. All these patients with ascites would have been candidates for genomic testing on cfDNA.

Ascites is frequent at diagnosis and relapse and yields large amounts of tumoral cfDNA. Our study demonstrates the feasibility of detecting somatic mutations in ascites of patients with high-grade OC. We provides a proof of concept that the measurement of genomic instability on cfDNA from ascites is feasible, detecting the same HRD scar as tumor testing. Our study has limitations. Our assay provides a rough measure of genomic instability and has not been validated against standard of care genomic instability tests. Although the cohort is small and the sampling heterogeneous, the exploratory results of our pilot study are very encouraging.

Very few data have been published on ctDNA in peritoneal fluid from patients with ovarian cancer. While standardization of assays remain to be addressed, this area of ​​study may help improve the management of patients with OC in optimizing early detection, therapeutic choices and monitoring of drug resistance [6, 10].

When tumor biopsies are inaccessible or when the tumor tissue is insufficient, ascites could be an alternative to tumor sampling for HRD and BRCA testing. Our results should be validated in large prospective studies.

### Electronic supplementary material

Below is the link to the electronic supplementary material.

Supplementary Material 1

## Data Availability

All data generated or analysed during this study are included in this published article and its Additional information files.

## List of abbreviations

cfDNA: Cell-free DNA
GIS: Genomic instability score
HRD: Homologous-Recombination deficiency
NGS: Next-generation sequencing
OC: Ovarian cancer
SCNA: Somatic copy number alterations
TP53m: TP53 mutation
