# Supplementary material for: Circulating Tumor DNA from Ascites as an alternative to tumor sampling for genomic profiling in ovarian cancer patients
Source: Biomark Res. 2023 Oct 20;11:93. doi: 10.1186/s40364-023-00533-1 (PMC10588202; doi:10.1186/s40364-023-00533-1)
Supplement: Supplementary file 1 — Supplementary Material 1 [file 40364_2023_533_MOESM1_ESM.docx]

**SUPPLEMENTARY** **MATERIAL**

**Material and Methods:**

**Patient inclusion and sample collection:**

Patients enrolled in a prospective biological study (OvBIOMark, NCT03010124) consented to analysis of tumor and ascites obtained as part of routine diagnosis (Supplementary figure 1). The study has been authorized by Gustave Roussy Institutional Review Board.

A volume of 10ml of fresh ascites was subjected to double centrifugation prior to being frozen at -80°.

In an final feasibility phase, operative reports from 100 consecutive patients diagnosed with stage III/IV high grade OC who underwent diagnostic laparoscopies at the Institut Gustave Roussy between April 2017 and February 2021 were retrospectively reviewed to assess the prevalence and volume of ascites.

**DNA extraction from ascites and targeted Next Generation sequencing:**

CfDNA was extracted from 1-4 ml of double-centrifuged fresh ascites using QIAamp®Circulating Nucleic Acid Kit according to manufacturer's instruction. The library was prepared with Ion AmpliSeq™Cancer Hotspot Panel v2, designed to amplify 207 amplicons covering approximately 2,800 COSMIC mutations from 50 oncogenes and tumor suppressor genes (ThermoFisher) (Supplementary Table 1).  Ion GeneStudio S5 System was used for sequencing. Samples were evaluated for genomic alterations, including single nucleotide variants (SNVs), and insertions and deletions, using Torrent Suite™ Software v2.3 (ThermoFisher Scientific), after alignment to the hg19 (GRCh37) human reference genome.

#### As per routine clinical practice at the time these samples were collected, only germline BRCA testing was performed. **Comparative Genomic Hybridization (CGH)/SNP array to measure genomic instability score**

#### Somatic Copy Number Alterations (SCNAs) were determined using either the Agilent SurePrint G3 CGH Microarray (8 × 60k probes, Agilent technologies) (n = 15) or the OncoScan FFPE Assay Kit (335k probes, Thermo Fisher Scientific) (n = 2). The genomic instability score (GIS) was measured as the number of altered segments superior to 15 Mbp and inferior to chromosome arm, and adjusted to ploidy: (number of altered segments X absolute difference to *exact* ASCAT2-predicted ploidy of the longest segment per chromosome) / by the genome length. Samples were classified as high versus low genomic instability using 20 as a cut off.

**Supplementary Figure 1:** **Timing and number of ascitic samples collection.**

Abbreviations: N = number of samples

**Supplementary Table 1**: Next-generation sequencing panel

**Supplementary Table 2**: Patients characteristics and mutation profile

| **Patient number** | **Histology** | **Timing of sample collection** | **cfDNA ng** | **Pathogenic *TP53 mutation*** | **Other pathogenic mutation** | **ctDNA AF** | **GIS** | **NGS on tumor** | **Germline *BRCA1/2* mutation** |
| --- | --- | --- | --- | --- | --- | --- | --- | --- | --- |
| **1** | HGSOC | Dx Lap | 2108 | p.Arg213* | No | 60 | 84 | pArg213 | WT |
|  |  | NACT | 536 | p.Arg213* | No | 76 | NA |  |  |
| **2** | HGSOC | Dx Lap | 1236 | p.Y220C | No | 76 | 22 | No | WT |
|  |  | NACT | 1120 | p.Y220C | No | 60 | NA |  |  |
|  |  | NACT | 264 | p.Y220C | No | 59 | NA |  |  |
|  |  | NACT | 2108 | p.Y220C | No | 47 | NA |  |  |
| **3** | HGSOC | Dx Lap | 532 | WT | No | NA | 27 | No | *BRCA1m* |
| **4** | HGSOC | Dx Lap | 4448 | p.R248W | No | 58 | 22 | No | WT |
|  |  | NACT | 1492 | p.R248W | No | 26 | NA |  |  |
| **5** | LGSOC | Dx Lap | 108 | WT | KRAS p.Q61H | 12 | NA | No | NA |
| **6** | HGSOC | Dx Lap | 1356 | p.R273H | No | 70 | 42 | No | *BRCA1m* |
| **7** | HGSOC | Relapse | 3664 | Splice /saut exon 5 | No | 87 | 1 | No | WT |
| **8** | HGSOC | Dx Lap | 1316 | p.R273H | No | 66 | 31 | No | *BRCA1m* |
|  |  | NACT | 892 | p.R273H | No | 61 | NA |  |  |
| **9** | LGSOC | Relapse | 1364 | WT | KRAS p.G12V | 5 | NA | No | NA |
| **10** | HGSOC | Dx Lap | 1448 | p.G245V | No | 71 | 2 | No | WT |
| **11** | HGSOC | IDS | 160 | WT | No | NA | <1 | No | WT |
| **12** | HGSOC | Relapse | 112 | p.F109V | No | 15 | NA | No | WT |
|  |  | Relapse | 24 | p.F109V | No | 35 | NA |  |  |
| **13** | HGSOC | Relapse | 2128 | p.R282W | No | 82 | NA | No | WT |
| **14** | HGSOC | Relapse | 1500 | p.R175H | No | 79 | 24 | No | NA |
|  |  | Relapse | 484 | p.R175H | No | 66 | NA |  |  |
| **15** | HGSOC | Dx Lap | 1184 | p.Arg213* | No | 75 | 43 | No | *BRCA2m* |
| **16** | HGSOC | Dx Lap | 1704 | p.Glu286_Glu287del | No | 24 | 17 | Glu286_Glu287 | WT |
|  |  | NACT | 1964 | p.Glu286_Glu287del | No | 24 | NA |  |  |
| **17** | HGSOC | Relapse | 284 | p.S215I | No | 4 | 46 | No | *BRCA2m* |
| **18** | HGSOC | Dx Lap | 56 | WT | No | NA | 3 | No | WT |
| **19** | HGSOC | Dx Lap | 980 | p.G266V | KRAS p.G12D | 34 | 3,5 | No | WT |
| **20** | HGSOC | Relapse | 424 | p.Gln192fs | KRAS p.G13D | 77 | 38 | No | WT |
| **21** | HGSOC | Dx Lap | 5732 | WT | No | NA | 24 | No | WT |
| **22** | HGSOC | Relapse | 80 | p.V274A | No | 74 | NA | No | WT |
